# Supplementary material for: Mechanisms governing target search and binding dynamics of hypoxia-inducible factors
Source: eLife. 2022 Nov 2;11:e75064. doi: 10.7554/eLife.75064 (PMC9681212; doi:10.7554/eLife.75064)
Supplement: Figure 1—source data 1. [file elife-75064-fig1-data1.zip › Figure 1 - source data 1/Figure of all uncropped blots with relavent bands labeled.pdf]

Left multi-channel blot image shows that this gel was cut in half and each was blotted with a different antibody

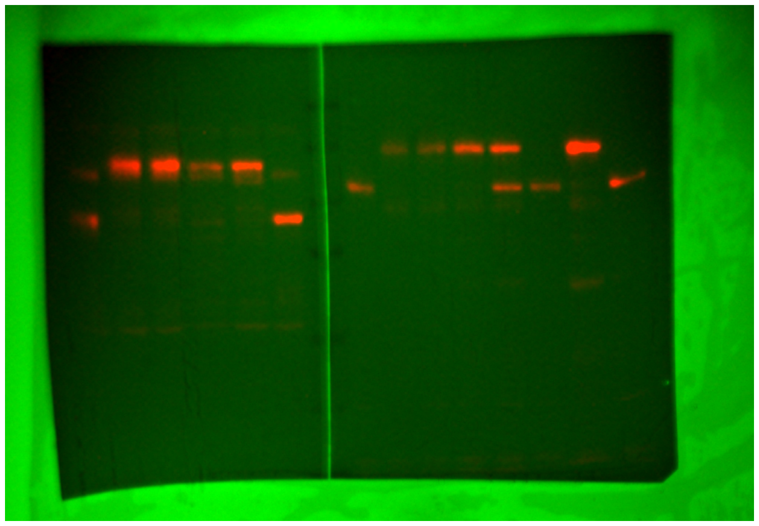

multichannel blot image showing anti-HIF1b and anti-HIF2a signal over the membrane

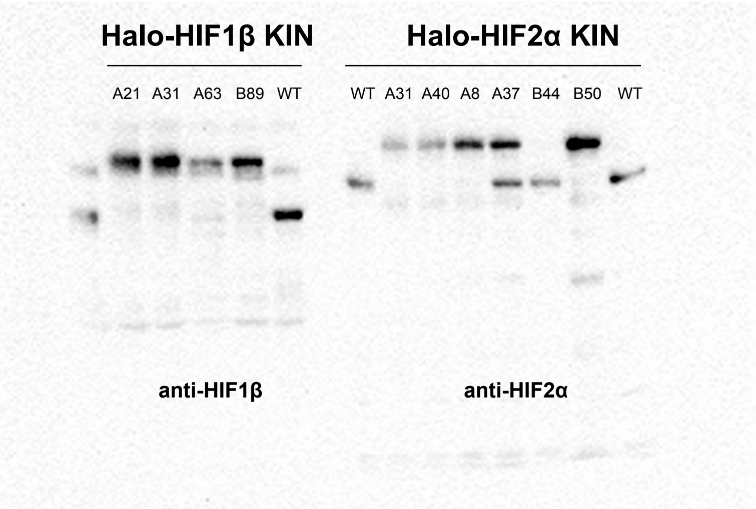

Original uncropped image for anti-HIF1b and anti-HIF2a signals

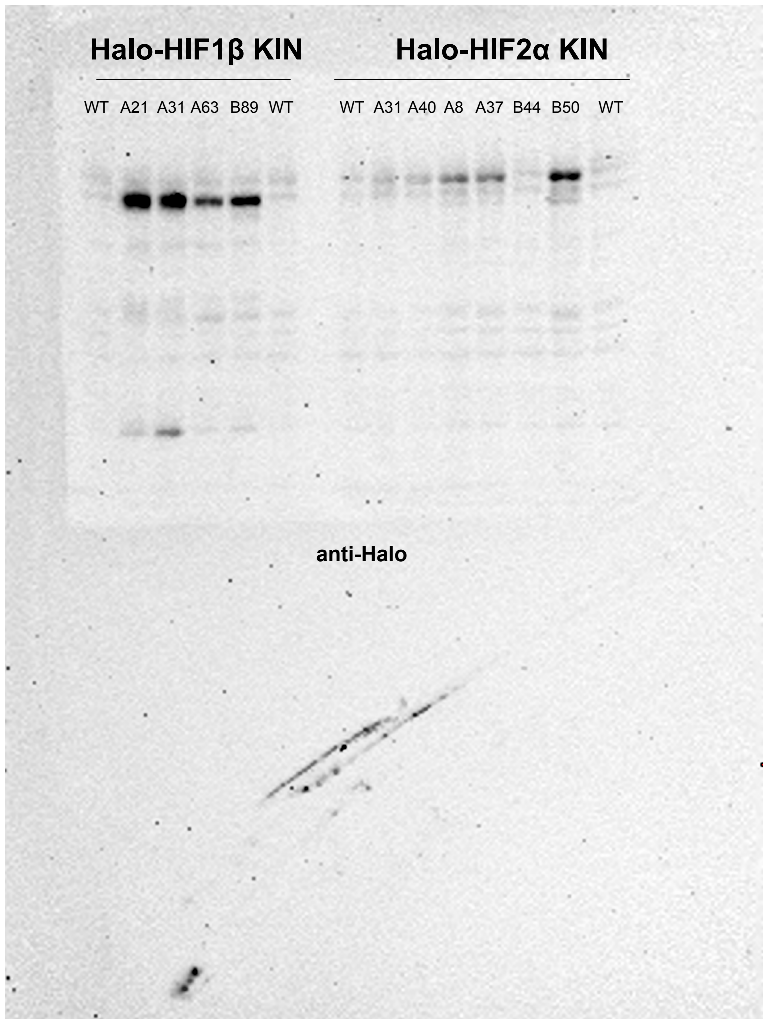

Original uncropped image for anti-Halo signals

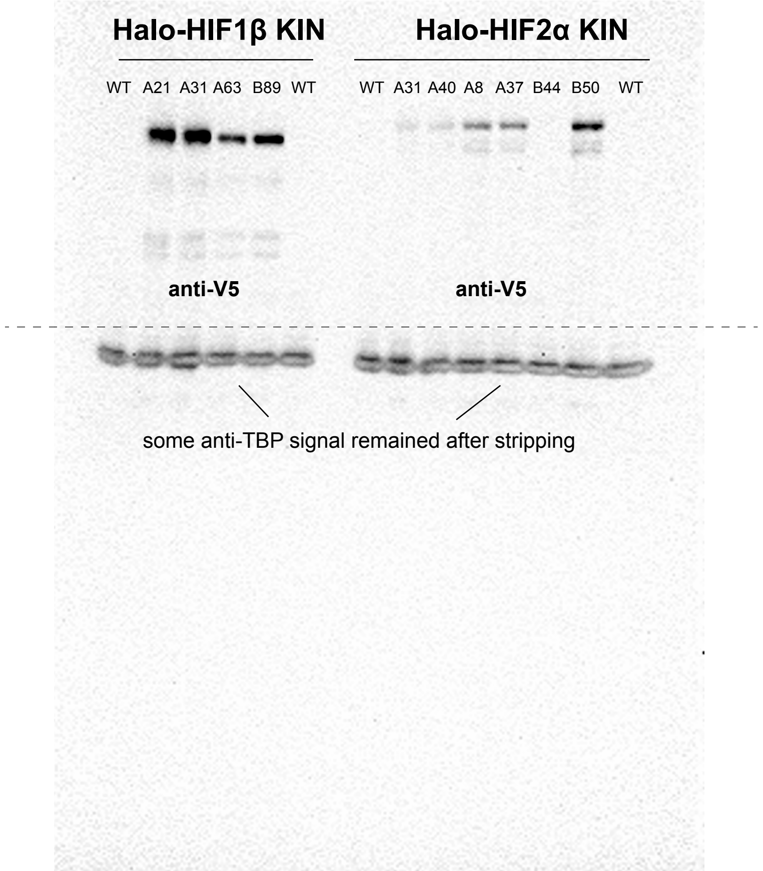

Original uncropped image for anti-V5 signals

Left multi-channel blot image shows TBP loading control for the three piece of membranes

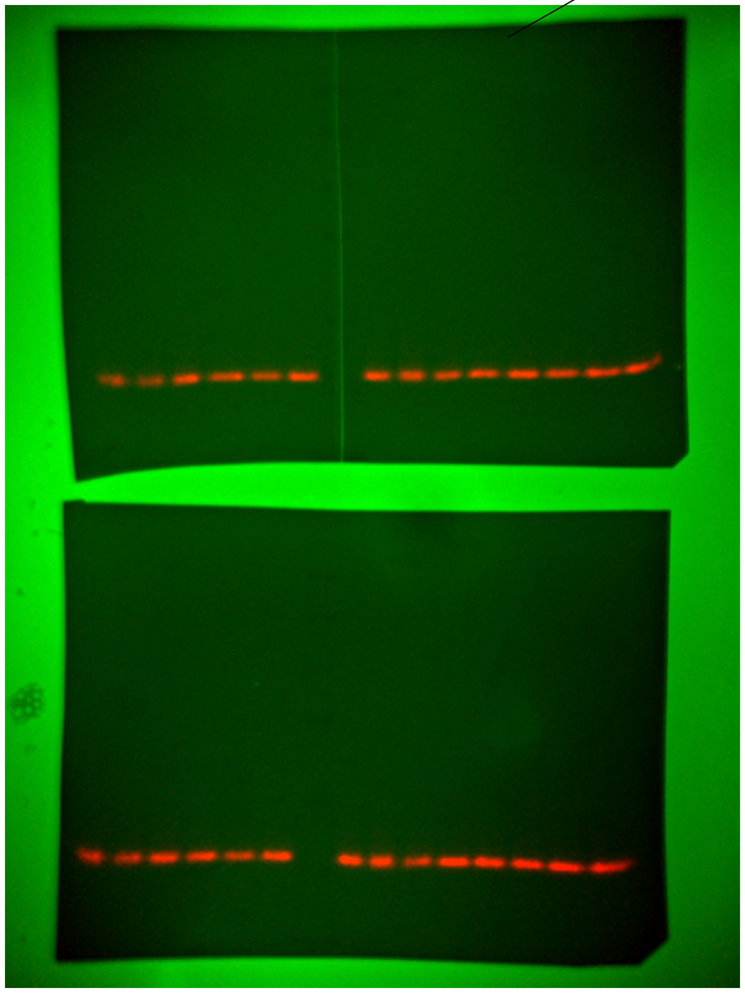

multichannel blot image showing anti-TBP singals over the membranes

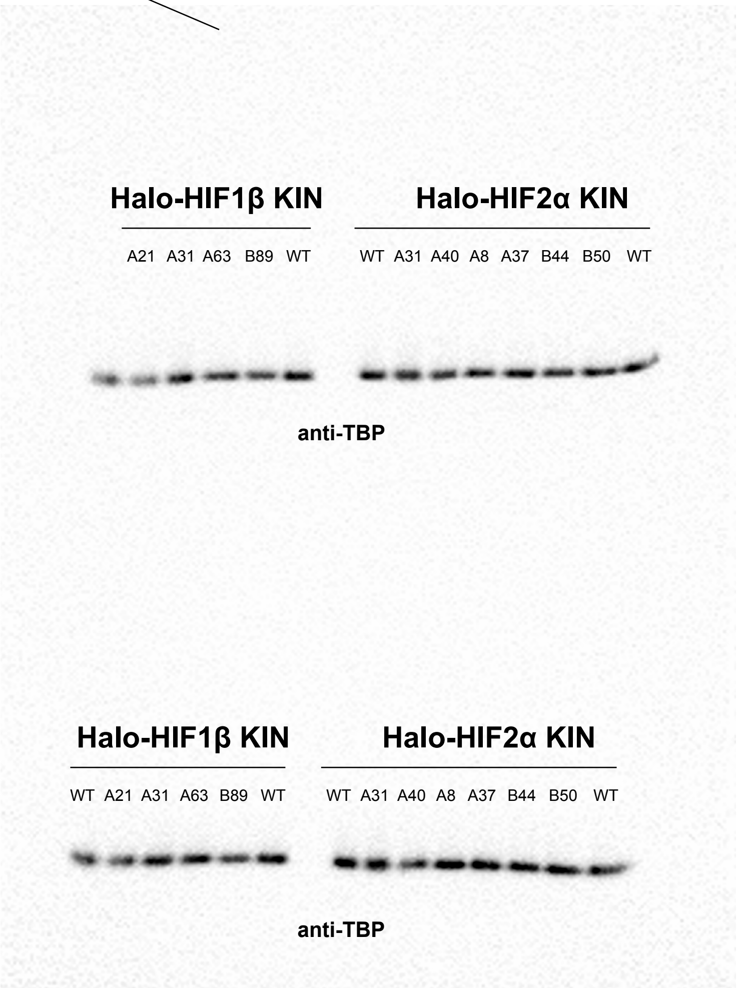

Original uncropped image for anti-TBP signals
