# Supplementary figures and images for: Mechanisms governing target search and binding dynamics of hypoxia-inducible factors
Source: eLife. 2022 Nov 2;11:e75064. doi: 10.7554/eLife.75064 (PMC9681212; doi:10.7554/eLife.75064)

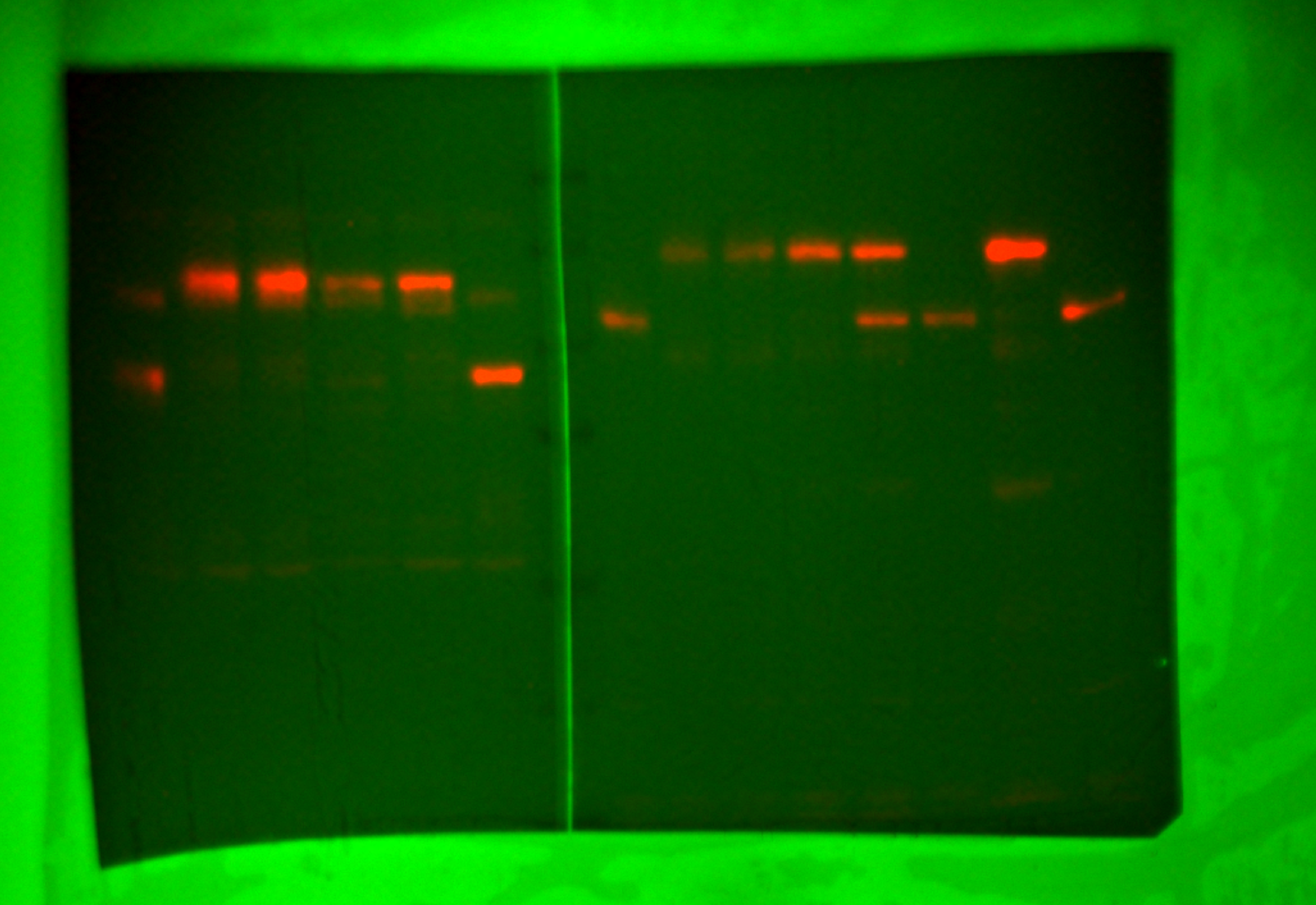

Supplement: Figure 1—source data 1. [file elife-75064-fig1-data1.zip › Figure 1 - source data 1/multichannel blot image showing anti-HIF1b and anti-HIF2a signal over the membrane.tif]

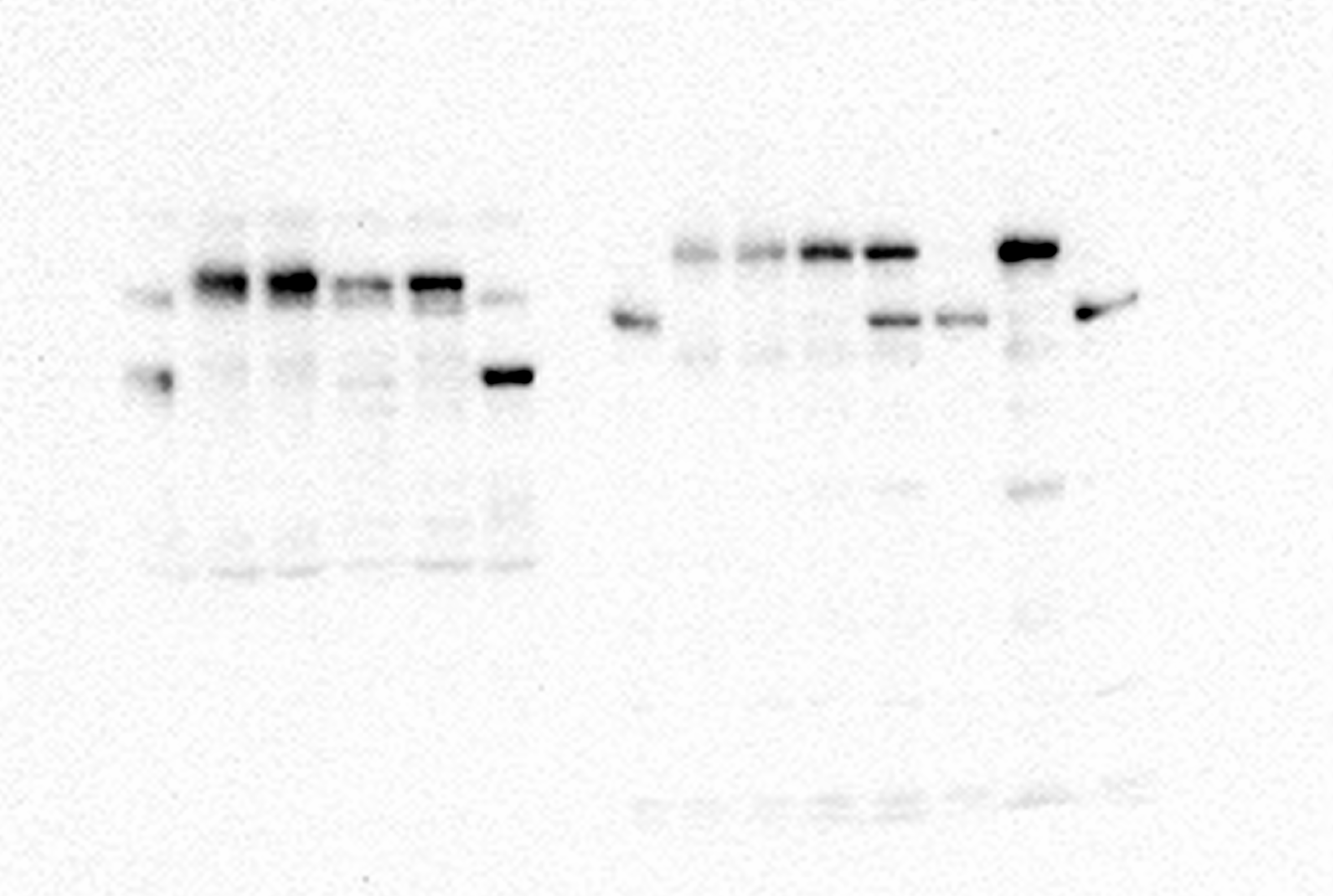

Supplement: Figure 1—source data 1. [file elife-75064-fig1-data1.zip › Figure 1 - source data 1/Original uncropped image for anti-HIF1b and anti-HIF2a signals.tif]

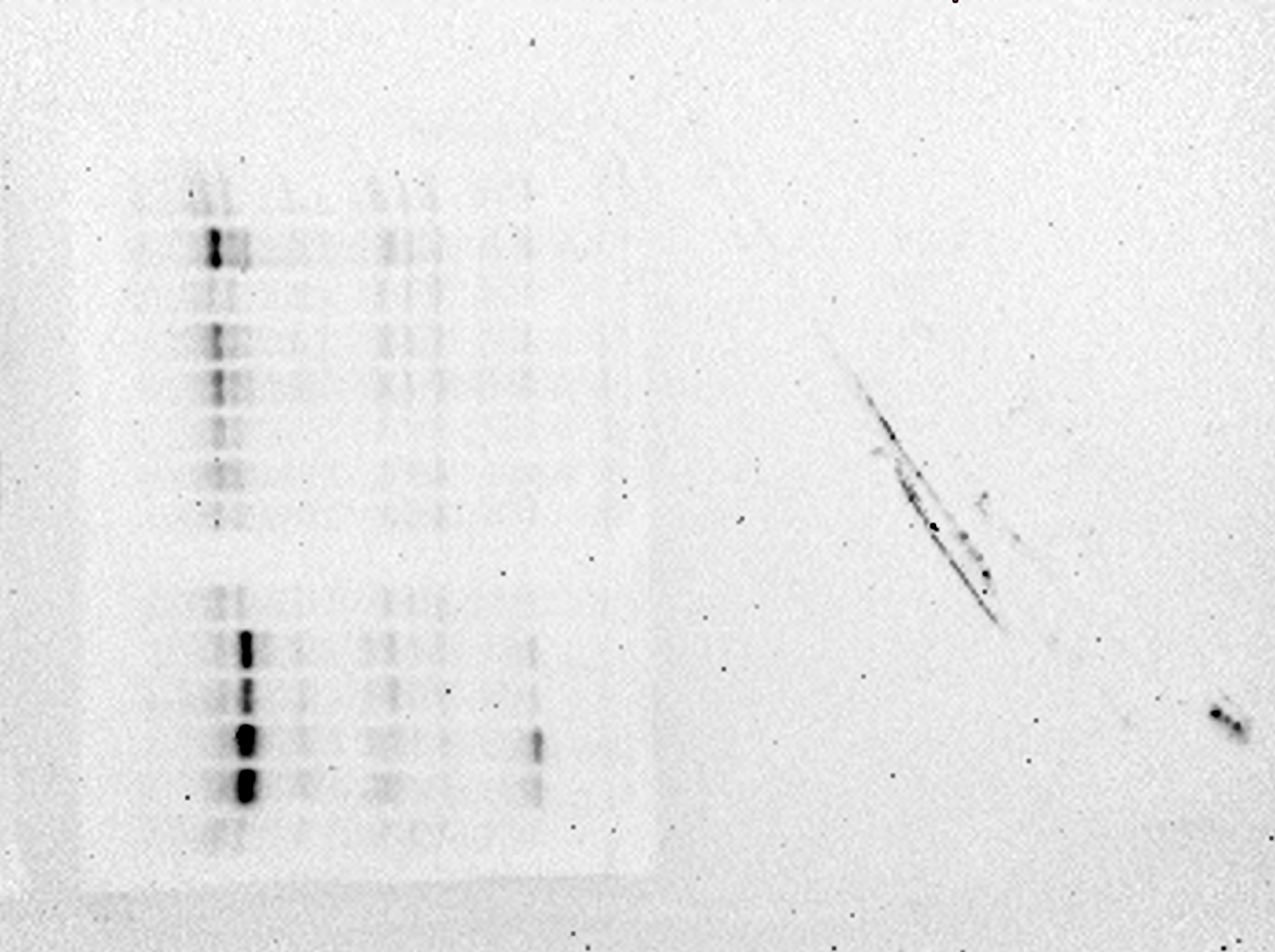

Supplement: Figure 1—source data 1. [file elife-75064-fig1-data1.zip › Figure 1 - source data 1/Original uncropped image for anti-Halo signals.tif]

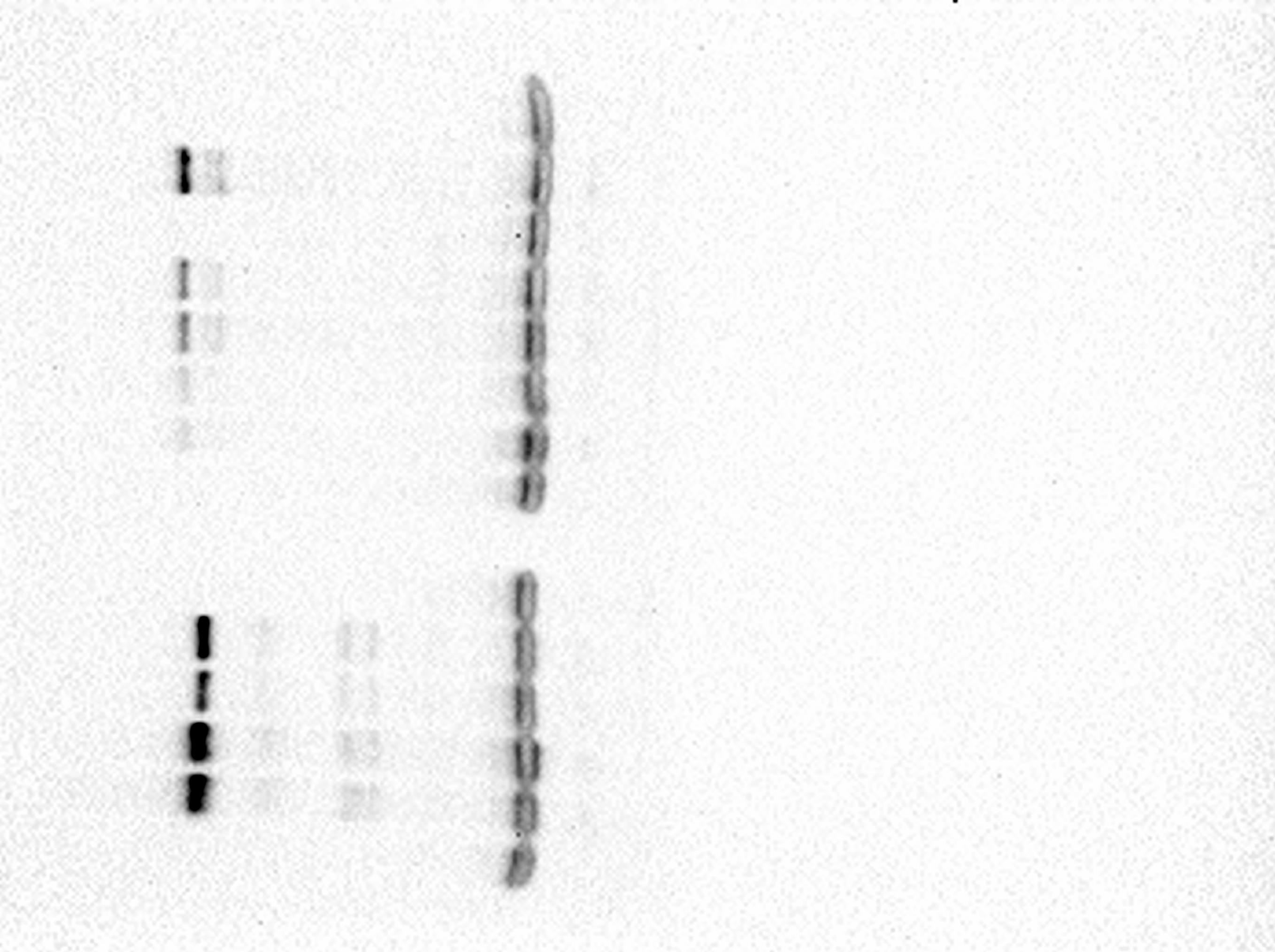

Supplement: Figure 1—source data 1. [file elife-75064-fig1-data1.zip › Figure 1 - source data 1/Original uncropped image for anti-V5 signals.tif]

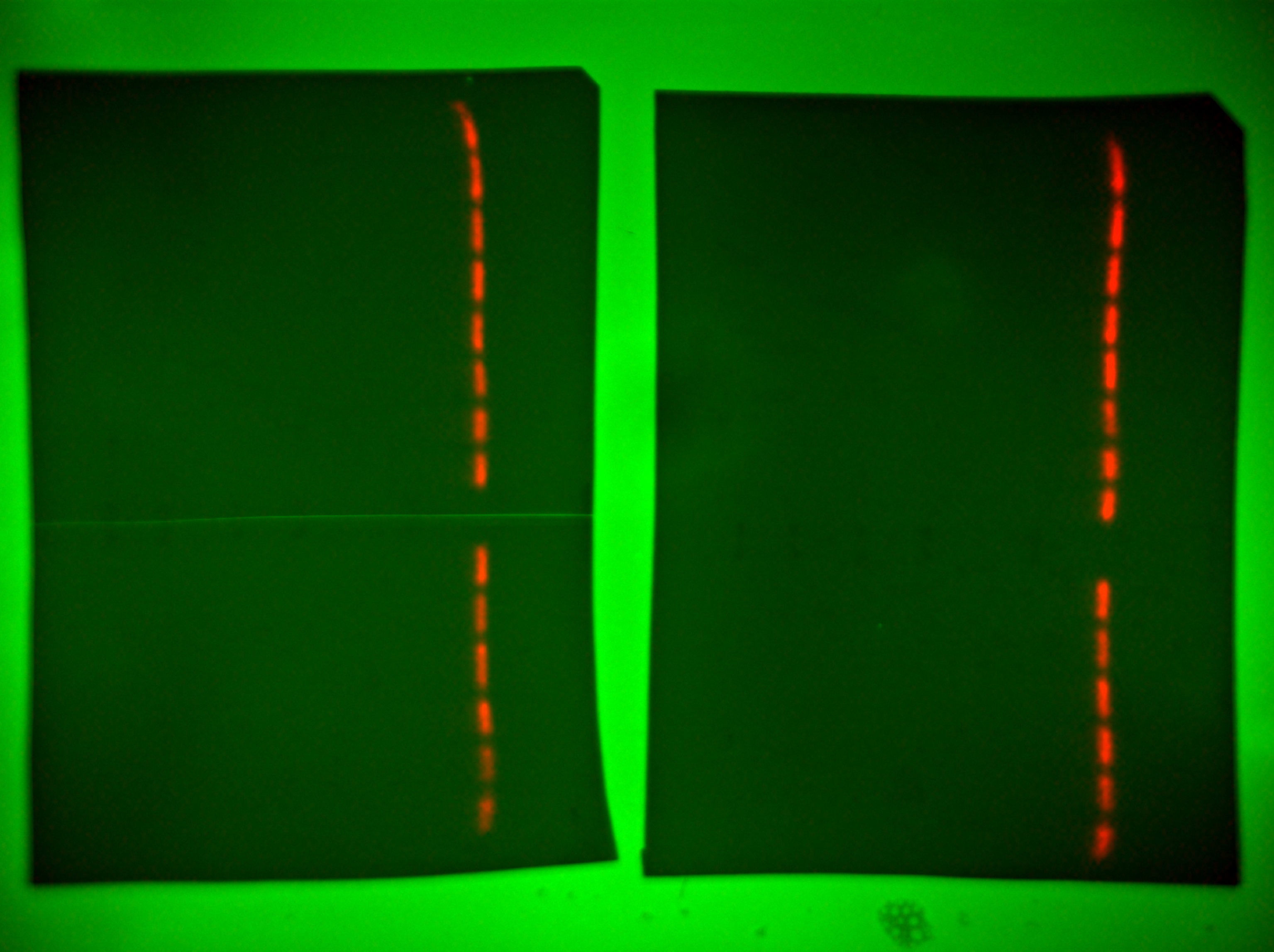

Supplement: Figure 1—source data 1. [file elife-75064-fig1-data1.zip › Figure 1 - source data 1/multichannel blot image showing anti-TBP singals over the membranes.tif]

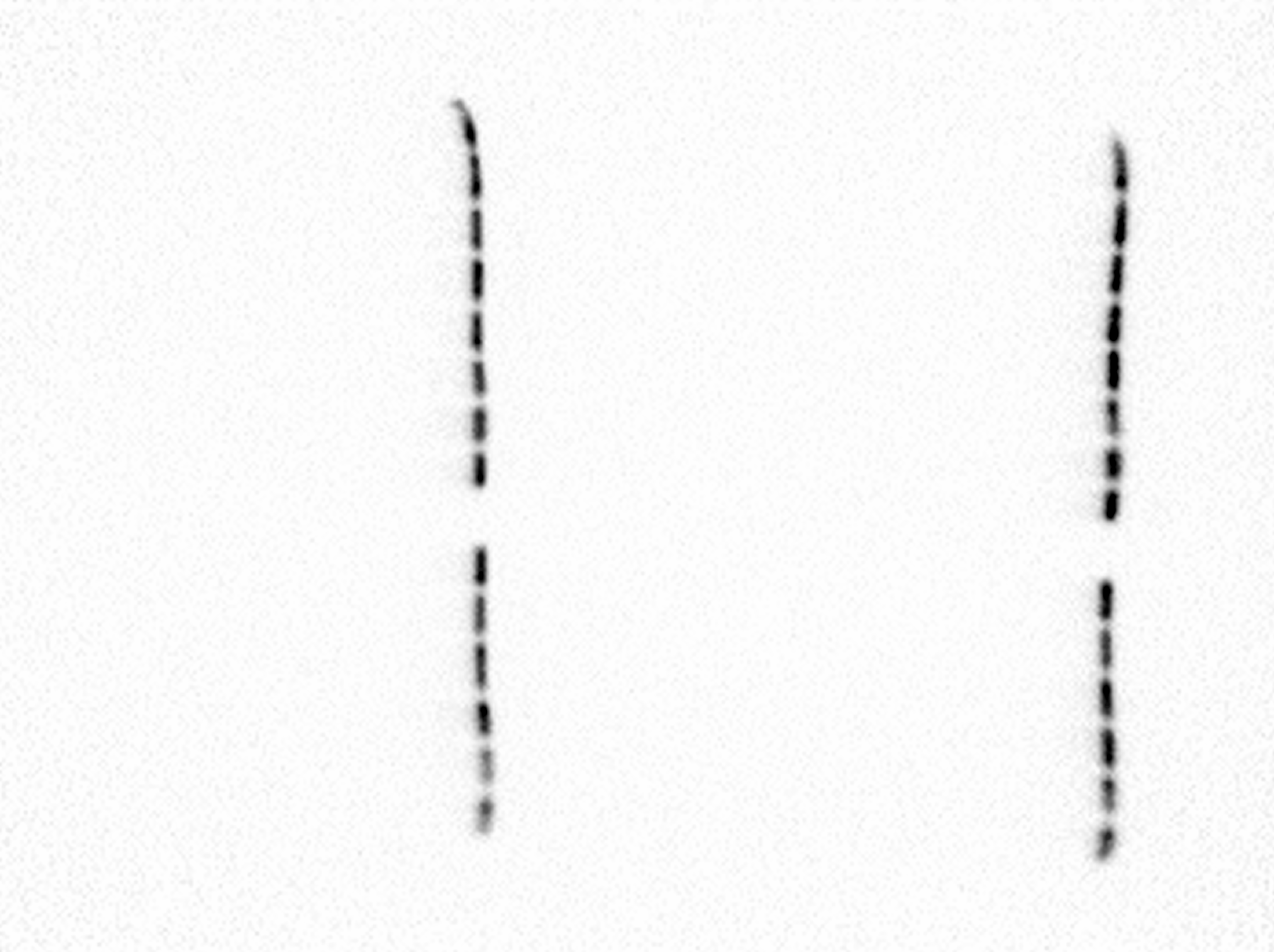

Supplement: Figure 1—source data 1. [file elife-75064-fig1-data1.zip › Figure 1 - source data 1/Original uncropped image for anti-TBP signals.tif]

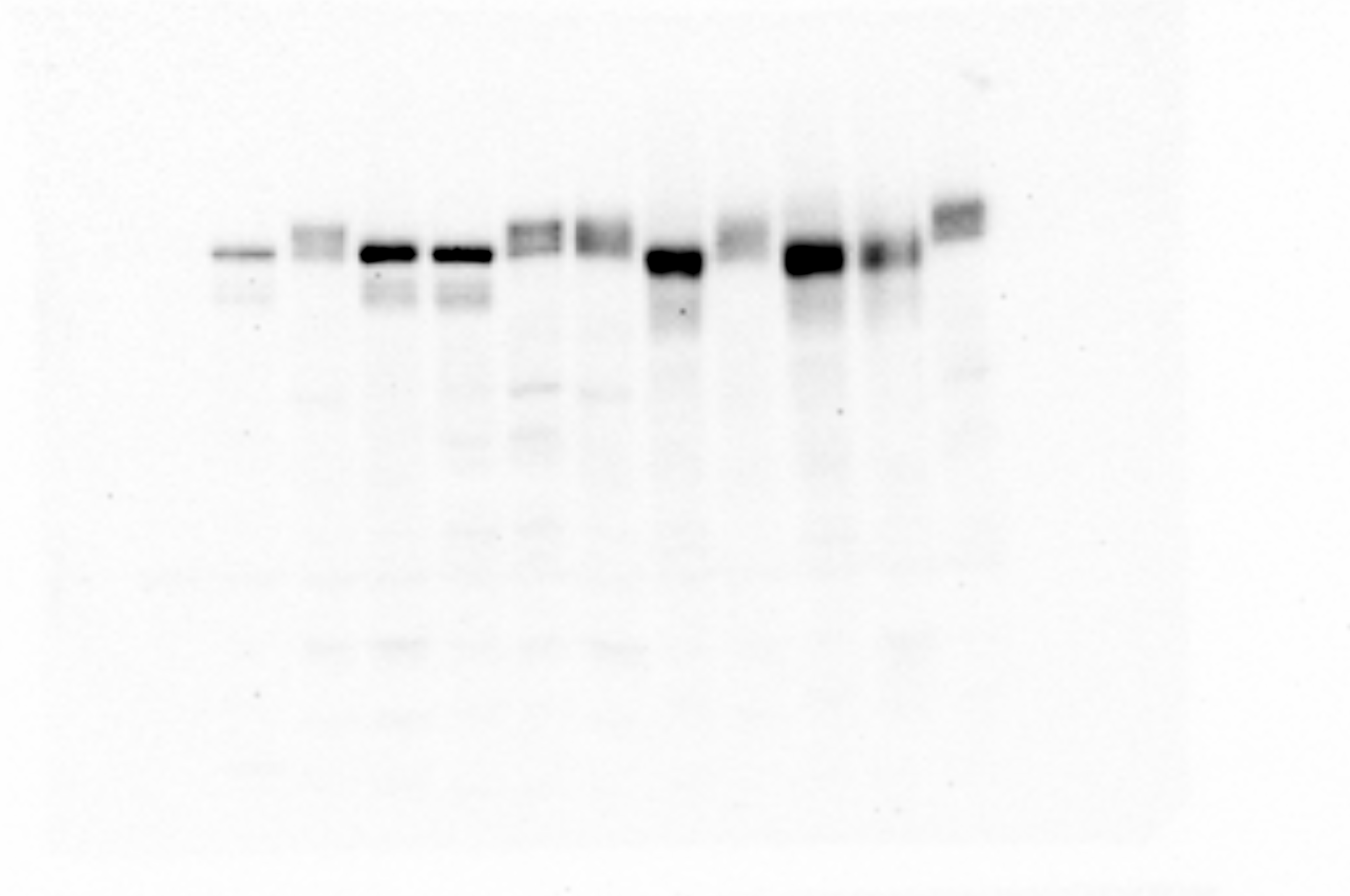

Supplement: Figure 4—figure supplement 1—source data 1. [file elife-75064-fig4-figsupp1-data1.zip › Figure4 - figure supplement 1 - source data 1/Original uncropped image for anti-V5 signals.tif]

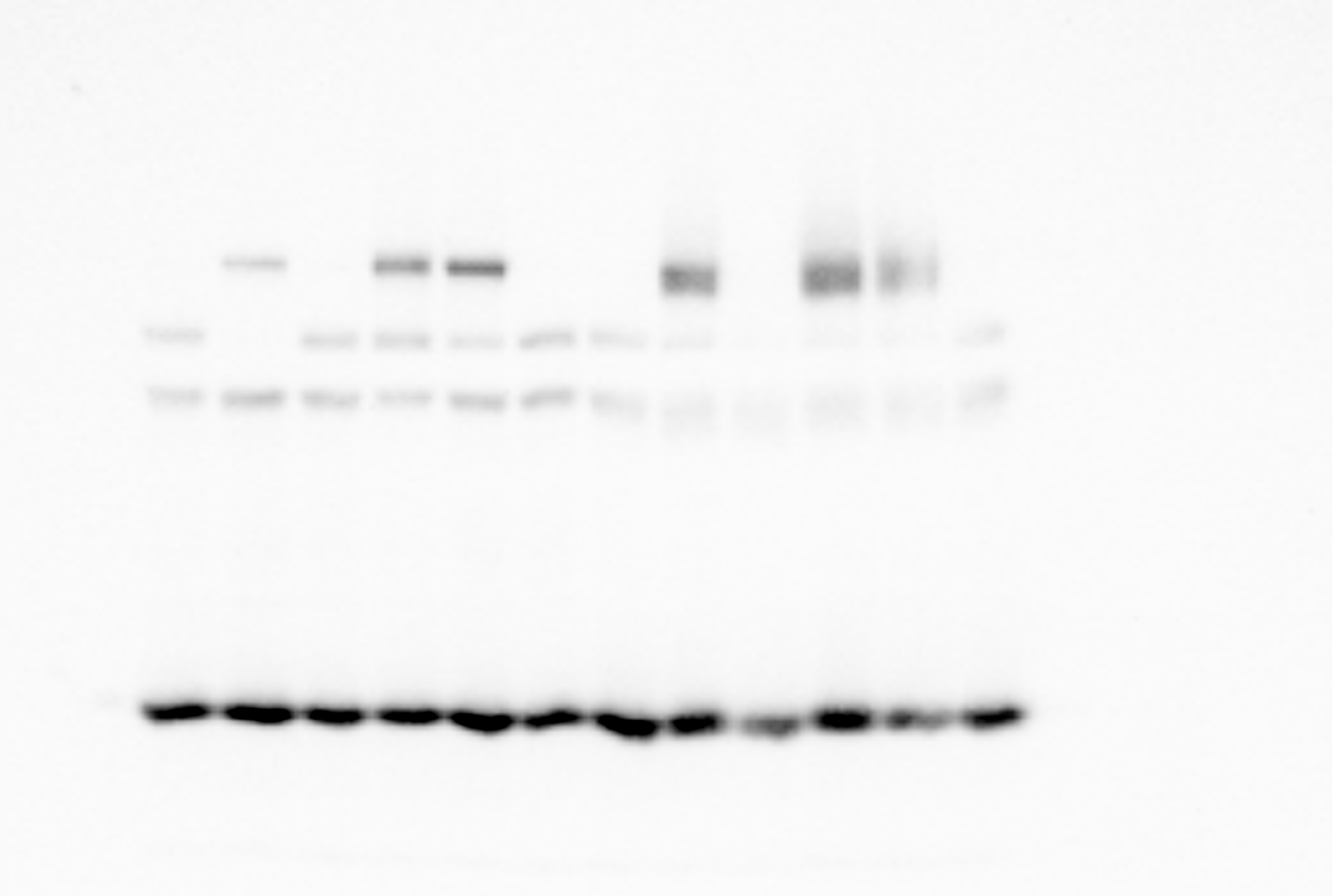

Supplement: Figure 4—figure supplement 1—source data 1. [file elife-75064-fig4-figsupp1-data1.zip › Figure4 - figure supplement 1 - source data 1/Original uncropped image for anti-TBP signals.tif]
