# Supplementary material for: Mechanisms governing target search and binding dynamics of hypoxia-inducible factors
Source: eLife. 2022 Nov 2;11:e75064. doi: 10.7554/eLife.75064 (PMC9681212; doi:10.7554/eLife.75064)
Supplement: Figure 4—figure supplement 1—source data 1. [file elife-75064-fig4-figsupp1-data1.zip › Figure4 - figure supplement 1 - source data 1/Figure of all uncropped blots with relavent bands labeled.pdf]

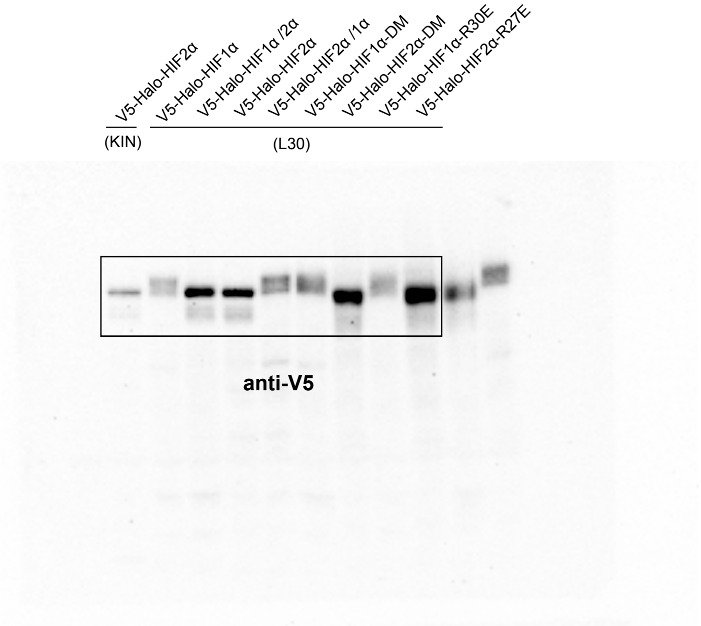

Original uncropped image for anti-V5 signals

same membrane reblotted with TBP antibody after stripping

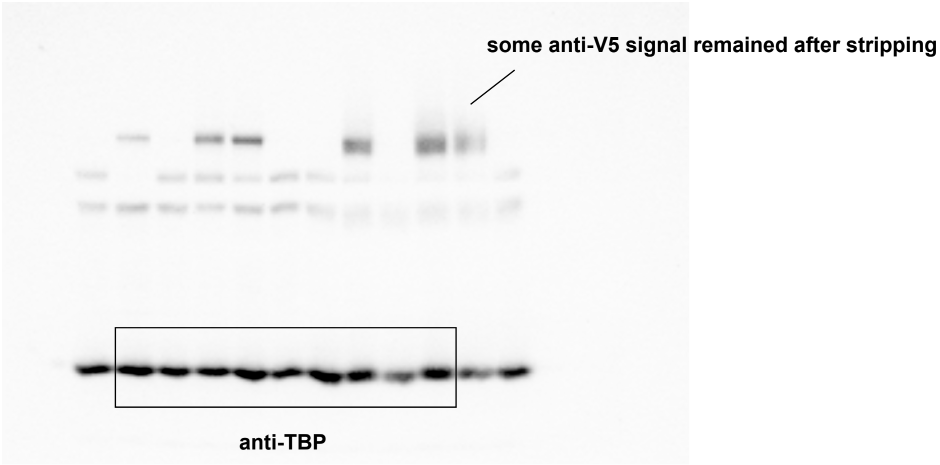

Original uncropped image for anti-TBP signals
